# First-line treatment with cadonilimab plus paclitaxel-platinum ± bevacizumab for persistent, recurrent, or metastatic cervical cancer: a retrospective real-world study

**DOI:** 10.3389/fonc.2025.1634779

**Published:** 2025-08-27

**Authors:** Xin Wang, Chunsheng Wang, Dan Zong, Biqing Zhu, Xia He

**Affiliations:** ^1^ Department of Radiotherapy, The Affiliated Cancer Hospital of Nanjing Medical University, Jiangsu Cancer Hospital, Jiangsu Institute of Cancer Research, Nanjing, China; ^2^ Collaborative Innovation Center for Cancer Personalized Medicine, Nanjing Medical University, Nanjing, China

**Keywords:** cadonilimab, immunotherapy, cervical cancer, progression-free survival, adverse events

## Abstract

**Background:**

The aim of this study was to assess the real-world effectiveness and safety of first-line treatment with cadonilimab plus paclitaxel-platinum ± bevacizumab for persistent, recurrent, or metastatic cervical cancer (p/r/m CC).

**Methods:**

In this retrospective real-world study from Jiangsu Cancer Hospital (January 2021–February 2025), patients with p/r/m CC received first-line cadonilimab plus paclitaxel-platinum ± bevacizumab or paclitaxel-platinum ± bevacizumab. Co-primary endpoints were progression-free survival (PFS) and safety; overall survival (OS), objective response rate (ORR), and disease control rate (DCR) were secondary. Kaplan–Meier and log-rank methods were applied, with prognostic factors analyzed using Cox models.

**Results:**

Among 169 eligible patients (50 cadonilimab plus TP; 119 TP), median follow-up was 33.2 months [interquartile range (IQR): 12.2–35.2]. Cadonilimab addition significantly prolonged mPFS [20.2 vs. 12.2 months; hazard ratio (HR): 0.531, *p* = 0.019], with 12- and 24-month PFS rates of 65.83% and 48.62% versus 50.71% and 29.57%, respectively. ORR improved from 40.3% to 54.0%, while DCR remained high in both cohorts (92.0% vs. 90.8%). mOS was not reached in the cadonilimab plus TP group and was 37.5 months with TP alone. Cadonilimab increased low-grade immune-related or gastrointestinal adverse events, with the most common being rash or itching (38.0%), pyrexia (32.0%), constipation (58.0%), and diarrhea (50.0%). However, events in grades 3–5 were infrequent. Subgroup analyses showed a generally consistent PFS benefit with cadonilimab across most predefined patient subsets.

**Conclusions:**

In real-world clinical settings, cadonilimab plus TP ± bevacizumab provides a durable PFS benefit with acceptable safety and supports first−line use for p/r/m CC; additional follow-up is essential to determine its impact on OS.

## 1 Introduction

Female-specific cancers such as breast, cervical, ovarian, and uterine cancer collectively account for approximately 40% of the overall cancer burden in women (1). Cervical cancer (CC) is one of the most common malignancies of the female reproductive system and ranks fourth globally in both incidence and mortality among women (2). Early-stage CC generally has a favorable prognosis; however, the 5-year survival rate for recurrent or metastatic cervical cancer (r/mCC) is less than 17% (3). Apart from platinum-based palliative chemotherapy, treatment options for patients with r/mCC remain limited and are primarily palliative in nature (4).

Based on the GOG-204 trial, cisplatin–paclitaxel has come to be recognized as the standard first-line regimen for r/m CC (5). The JCOG-0505 study further demonstrated that carboplatin plus paclitaxel may serve as an alternative for patients previously treated with or intolerant to cisplatin (6). Additionally, the GOG-240 study showed that adding bevacizumab to first-line chemotherapy confers a survival advantage (7). Consequently, the current standard first-line regimens typically consist of paclitaxel combined with either cisplatin or carboplatin ± bevacizumab (8). With the growing application of immunotherapy in CC, its indications have expanded from later-line to first-line settings. To date, three major phase III randomized controlled trials (RCTs) have reported results on first-line immunotherapy for r/m CC: KEYNOTE-826 (9), BEATcc (10), and AK104-303 (11).

Bispecific antibodies, engineered to concurrently engage dual immune checkpoints and/or signaling pathways within a single molecule, have emerged as a promising strategy to enhance synergistic antitumor activity. Beyond PD−1/CTLA−4 constructs, ivonescimab (AK112) co−targets PD−1 and VEGF−A, illustrating the rationale for cross−pathway bispecific antibodies (12). Similarly, Yi et al. (13) demonstrated that a PD−L1/TGF−β bispecific antibody can concurrently potentiate antitumor immunity and reprogram an immunosuppressive tumor microenvironment.

Cadonilimab is a first-in-class bispecific that simultaneously engages PD-1 and CTLA-4 and has been authorized in China for r/m CC after progression on platinum-based therapy (14). AK104-303, a phase III, randomized, double-blind, multicenter study conducted in China, assessed cadonilimab plus paclitaxel-platinum ± bevacizumab as initial systemic treatment for p/r/m CC. Adding cadonilimab to chemotherapy significantly improved progression-free survival (PFS) and overall survival (OS), with acceptable tolerability: mPFS, 12.7 vs. 8.1 months [hazard ratio (HR) = 0.62; 95% confidence interval (CI): 0.49–0.80; *p* < 0.0001], and mOS, not reached vs. 22.8 months (HR = 0.64; 95% CI: 0.48–0.86; *p* = 0.0011).

While RCTs are designed with strict inclusion criteria and aim to minimize confounding by limiting non-study medications, they may not fully reflect real-world clinical practice (15, 16). Evidence regarding the real-world effectiveness, safety, maintenance strategies, and integration with radiotherapy of front-line immunotherapy remains limited. Recent reviews (17, 18) have underscored the need to complement randomized data with real-world outcomes to optimize patient selection and therapeutic decision-making. Moreover, real-world evidence regarding the use of cadonilimab in CC remains scarce, and its effectiveness and safety require validation in real-world clinical settings.

Therefore, we retrospectively assessed real-world effectiveness and safety outcomes of first-line treatment with cadonilimab plus paclitaxel-platinum ± bevacizumab for p/r/m CC.

## 2 Materials and methods

### 2.1 Patients

From January 2021 to February 2025, patients with p/r/m CC who received first-line cadonilimab plus paclitaxel-platinum ± bevacizumab or paclitaxel-platinum ± bevacizumab alone at the Department of Gynecologic Radiation Oncology, Jiangsu Cancer Hospital, were enrolled in the study.

**Figure 1:**
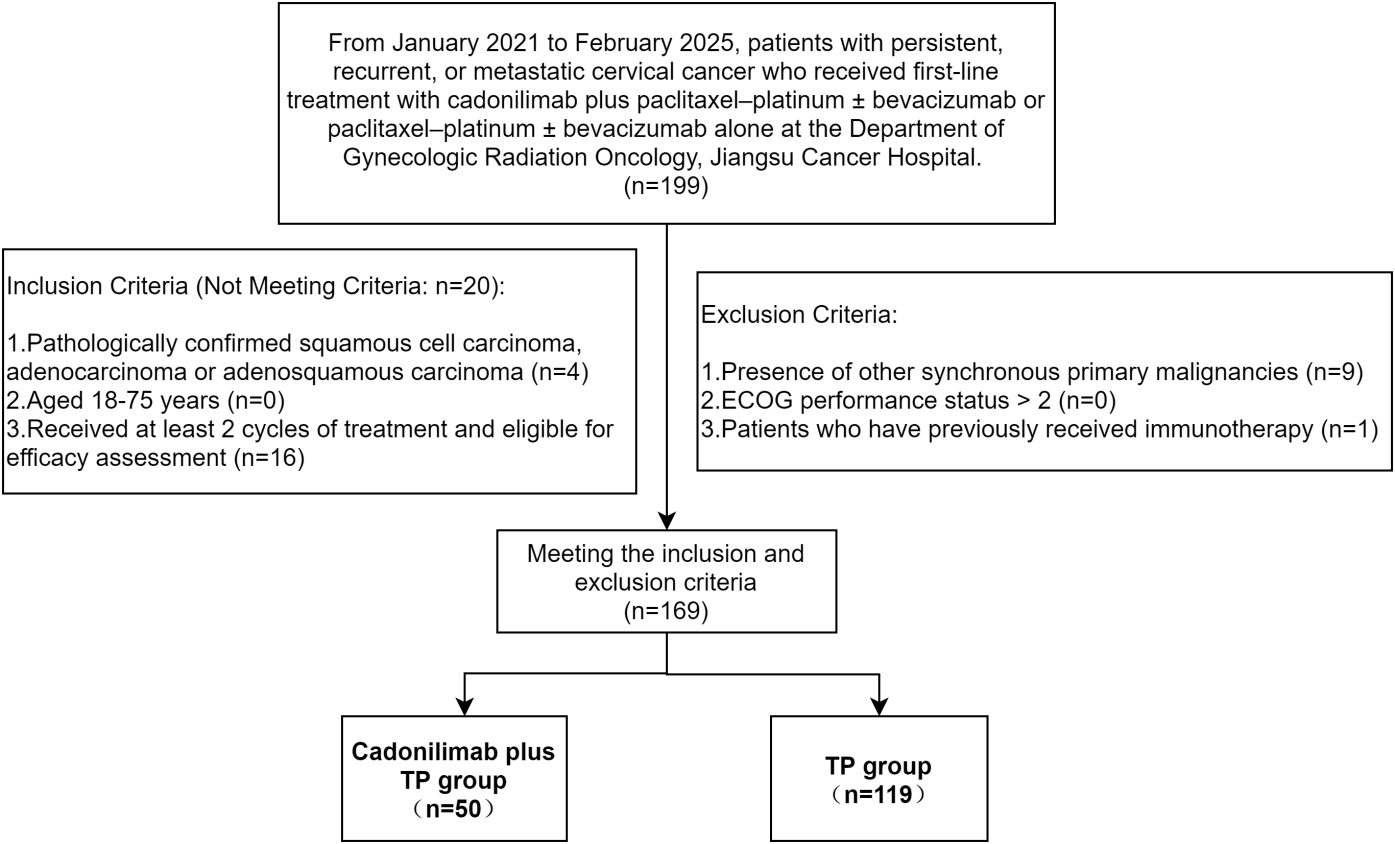
Flowchart of patient inclusion and exclusion criteria.

The inclusion and exclusion criteria are summarized in **Figure 1**. Notably, cadonilimab was first approved for use in mainland China on 29 June 2022; therefore, all patients in the cadonilimab plus TP group were enrolled after this date.

Following exclusion of 30 patients, 169 were analyzed. The Ethics Committee of Jiangsu Cancer Hospital approved the protocol (KY-2025-067) and waived informed consent due to the retrospective design.

### 2.2 Treatment protocol

One of the following formulations was administered: paclitaxel (135–175 mg/m²), paclitaxel liposome (135–175 mg/m²), or albumin-bound paclitaxel (260 mg/m²). When appropriate, docetaxel (60–75 mg/m²) was administered as a substitute. The taxane was combined with cisplatin (50 mg/m²) or, alternatively, with carboplatin (AUC 4–5), both given via intravenous infusion, q3w. This combination constituted the TP regimen, which was administered with or without concurrent bevacizumab (15 mg/kg, intravenous infusion, q3w). Chemotherapy dosages were adjusted as needed according to the patient’s general condition and treatment tolerance.

Cadonilimab (AK104; manufactured by Akeso Biopharma Inc., Zhongshan, China) was administered intravenously at a dose of 10 mg/kg, q3w in the cadonilimab plus TP group.

Radiotherapy was administered at the discretion of the treating radiation oncologist, with treatment parameters tailored based on lesion characteristics, including metastatic or recurrent site, tumor volume, and surrounding organ constraints.

Maintenance therapy strategies were guided by multidisciplinary consensus, considering radiographic response assessments and patient preference. These strategies encompassed a range of approaches, including immunotherapy monotherapy (either a PD-1 inhibitor or cadonilimab q3w), targeted therapy maintenance (bevacizumab q3w or oral anlotinib 8–12 mg on days 1–14 of each q3w cycle), and combination regimens involving both immunotherapy and targeted agents. The duration of maintenance therapy was individualized based on multiple factors, including disease stability, patient tolerance, clinician judgment, and patient preference.

### 2.3 Outcome assessment and safety evaluation

After treatment completion, patients underwent surveillance every 3 months during the initial 2 years, with final follow-up data censored in June 2025.

Response after two cycles was determined by pelvic magnetic resonance imaging (MRI; non-contrast- and contrast-enhanced). Chest and abdominal computed tomography (CT) scans with identical contrast enhancement protocols were also performed. Responses were CR (complete response)/PR (partial response)/SD (stable disease)/PD (progressive disease); objective response rate (ORR) = CR + PR and disease control rate (DCR) = CR + PR + SD (RECIST v1.1).

Additionally, in the cadonilimab plus TP group, the best overall confirmed response was evaluated. We generated a waterfall plot displaying each patient’s maximal percent change from baseline in the aggregate diameter of target lesions, with the corresponding confirmed best overall response (BOR) annotated. A swim-lane plot was constructed to illustrate the follow-up duration for each patient, including the time to first CR/PR/SD/PD, death, and last follow-up date. The background color of each bar denoted the confirmed BOR.

PFS was defined as the time from treatment initiation to either documented disease progression or death from any cause. OS was defined as the time from treatment initiation to death from any cause. Treatment-related adverse events (TRAEs) were graded by NCI-CTCAE v5.0 (19).

### 2.4 Statistical analysis

Analyses and figure generation were performed in SPSS (v29.0) and R (v4.5.1). Categorical data are presented as counts and percentages (*n*, %), while continuous measures are summarized as mean (SD). Between-group differences for categorical variables were assessed using Pearson’s *χ*² or Fisher’s exact test, as appropriate. Kaplan–Meier methods were used to plot survival, and groups were compared with the log-rank test. Cox proportional-hazards models were applied to explore prognostic factors for PFS and OS; covariates with *p* < 0.05 in univariable analyses were entered into multivariable models. All hypothesis tests were two-tailed, with *p* < 0.05 indicating statistical significance.

## 3 Results

### 3.1 Baseline characteristics

A total of 169 patients were included in the study, with 119 receiving first-line treatment with paclitaxel-platinum ± bevacizumab (TP group) and 50 receiving cadonilimab combined with paclitaxel-platinum ± bevacizumab (cadonilimab plus TP group). Baseline characteristics were comparable across groups (**Table 1**).

**Table 1:** Patient characteristics (*N* = 169).

Characteristic	TP group (*n* = 119), *N* (%)	Cadonilimab plus TP group (*n* = 50), *N* (%)	*χ* ^2^	*p*-value
**Age**			0.002	0.961
<65	102 (85.7)	43 (86.0)		
≥65	17 (14.3)	7 (14.0)		
**Diabetes**			1.529	0.216
Yes	21 (17.6)	13 (26.0)		
No	98 (82.4)	37 (74.0)		
**Hypertension**			2.840	0.092
Yes	32 (26.9)	20 (40.0)		
No	87 (73.1)	30 (60.0)		
**Pathological diagnosis**			0.017	0.898
Squamous cell carcinoma	99 (83.2)	42 (84.0)		
Adenocarcinoma	20 (16.8)	8 (16.0)		
**Histologic grade**			2.443	0.118
1, 2, x	68 (57.1)	22 (44.0)		
3	51 (42.9)	28 (56.0)		
**ECOG PS**			1.253	0.534
0	28 (23.5)	11 (22.0)		
1	67 (56.3)	25 (50.0)		
2	24 (20.0)	14 (28.0)		
**Disease status**			6.820	0.033
Metastatic (FIGO 2018: IVB)	38 (31.9)	14 (28.0)		
Persistent or recurrent with distant metastases	56 (47.1)	16 (32.0)		
Persistent or recurrent without distant metastases	25 (21.0)	20 (40.0)		
**Recurrence-free interval**			0.016	0.900
≤1 year	63 (52.9)	27 (54.0)		
>1 year	56 (47.1)	23 (46.0)		
**Lymph node metastasis**			0.061	0.805
Yes	81 (68.1)	35 (70.0)		
No	38 (31.8)	15 (30.0)		
**Lung metastasis**			3.046	0.081
Yes	37 (31.1)	9 (18.0)		
No	82 (68.9)	41 (82.0)		
**Pelvic cavity metastasis**			9.318	0.002
Yes	39 (32.8)	29 (58.0)		
No	80 (67.2)	21 (42.0)		
**Bone metastasis**			2.216	0.137
Yes	23 (19.3)	5 (10.0)		
No	96 (80.7)	45 (90.0)		
**Liver metastasis**			——	1.000
Yes	7 (5.9)	2 (4.0)		
No	112 (94.1)	48 (96.0)		
**Metastatic region count**			0.512	0.474
Single	50 (42.0)	24 (48.0)		
Multiple	69 (58.0)	26 (52.0)		
**Bevacizumab use during the treatment**			0.981	0.322
Yes	45 (37.8)	23 (46.0)		
No	74 (62.2)	27 (54.0)		
**Radiotherapy for recurrence**			0.920	0.337
Yes	76 (63.9)	28 (56.0)		
No	43 (36.1)	22 (44.0)		
**Maintenance therapy**			23.129	<0.001
Yes	26 (21.8)	30 (60.0)		
No	93 (78.2)	20 (40.0)		

For 2×2 contingency table variables with expected cell counts less than 5 (e.g., liver metastasis), Fisher’s exact test was used instead of the chi-square test, and the chi-square value was not reported.

Bold values are shown solely for visual clarity and do not carry any statistical implication.

A majority were younger than 65 years: 102 patients (85.7%) in the TP group and 43 (86.0%) in the cadonilimab plus TP group. ECOG PS 0–1 was documented in 95 (79.8%) patients in the TP group and 36 (72.0%) patients in the cadonilimab plus TP group. Squamous cell carcinoma was the predominant pathological subtype in both groups (83.2% vs. 84.0%).

A total of 38 patients in the TP group and 14 patients in the cadonilimab plus TP group were initially diagnosed with metastatic disease (FIGO 2018: IVB). Bevacizumab was administered to 45 patients (37.8%) in the TP group and 23 patients (46.0%) in the cadonilimab plus TP group. Additionally, 76 patients (63.9%) in the TP group and 28 patients (56.0%) in the cadonilimab plus TP group received regional radiotherapy targeting recurrent or metastatic lesions.

Relative to the TP group, the cadonilimab plus TP group showed a greater proportion of pelvic cavity metastasis (58.0% vs. 32.8%, *p* = 0.002) and was more likely to receive maintenance therapy (60.0% vs. 21.8%, *p* < 0.001).

### 3.2 Best overall response in the cadonilimab plus TP group

Within the cadonilimab plus TP group, patients received a median of 5.2 treatment cycles (range: 3–8). We conducted a dedicated analysis of the confirmed BOR.

**Figure 2:**
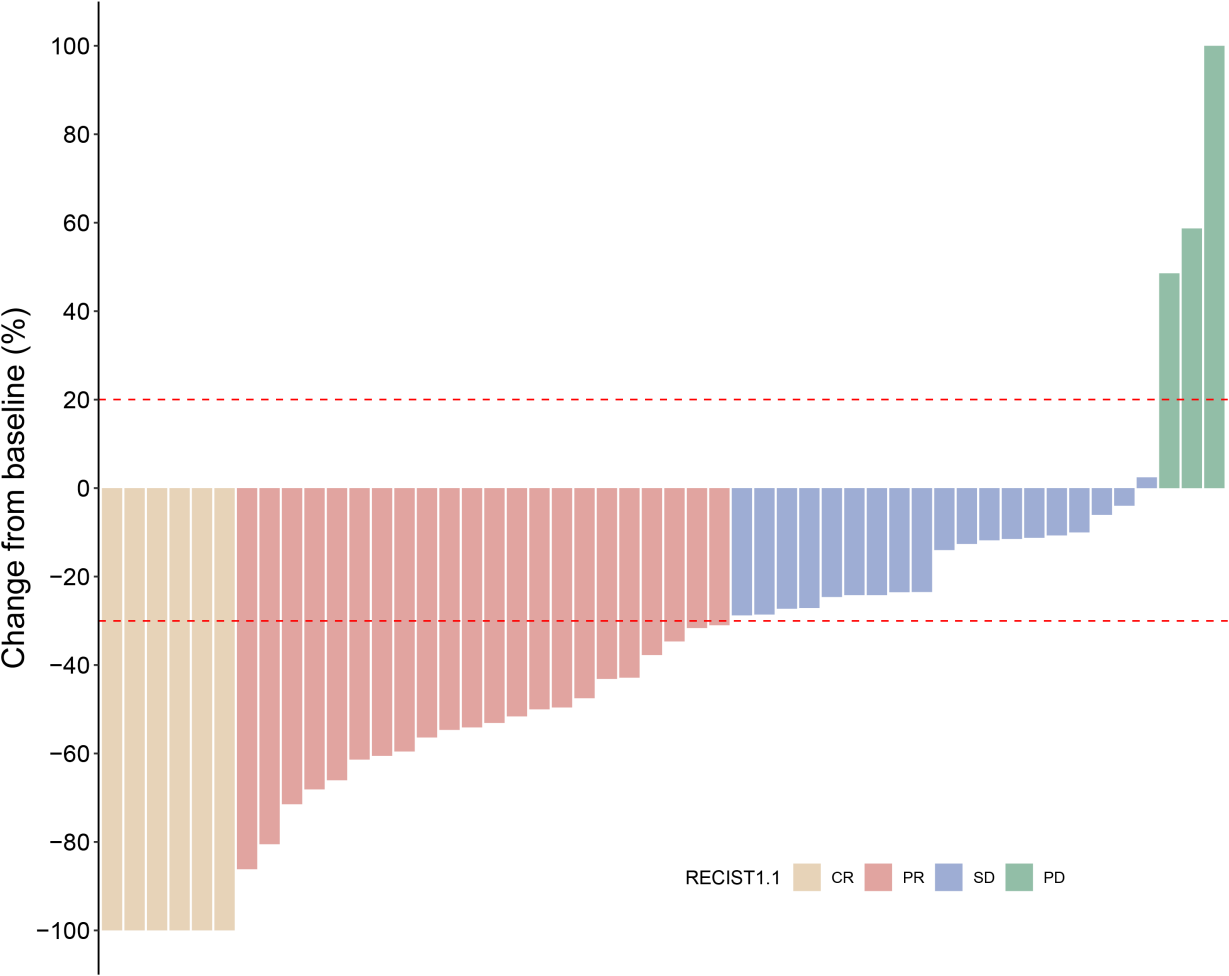
Waterfall plot of the best percentage of change from baseline in sum of diameters with best overall confirmed response (*N* = 50).

Among the 50 evaluable patients, BOR is summarized in **Figure 2**. Six patients (12.0%) achieved a CR and 22 (44.0%) achieved a PR, yielding an ORR of 56.0%. A further 19 patients (38.0%) experienced SD, resulting in a DCR of 94.0%. PD as the best response was documented in three patients (6.0%).

**Figure 3:**
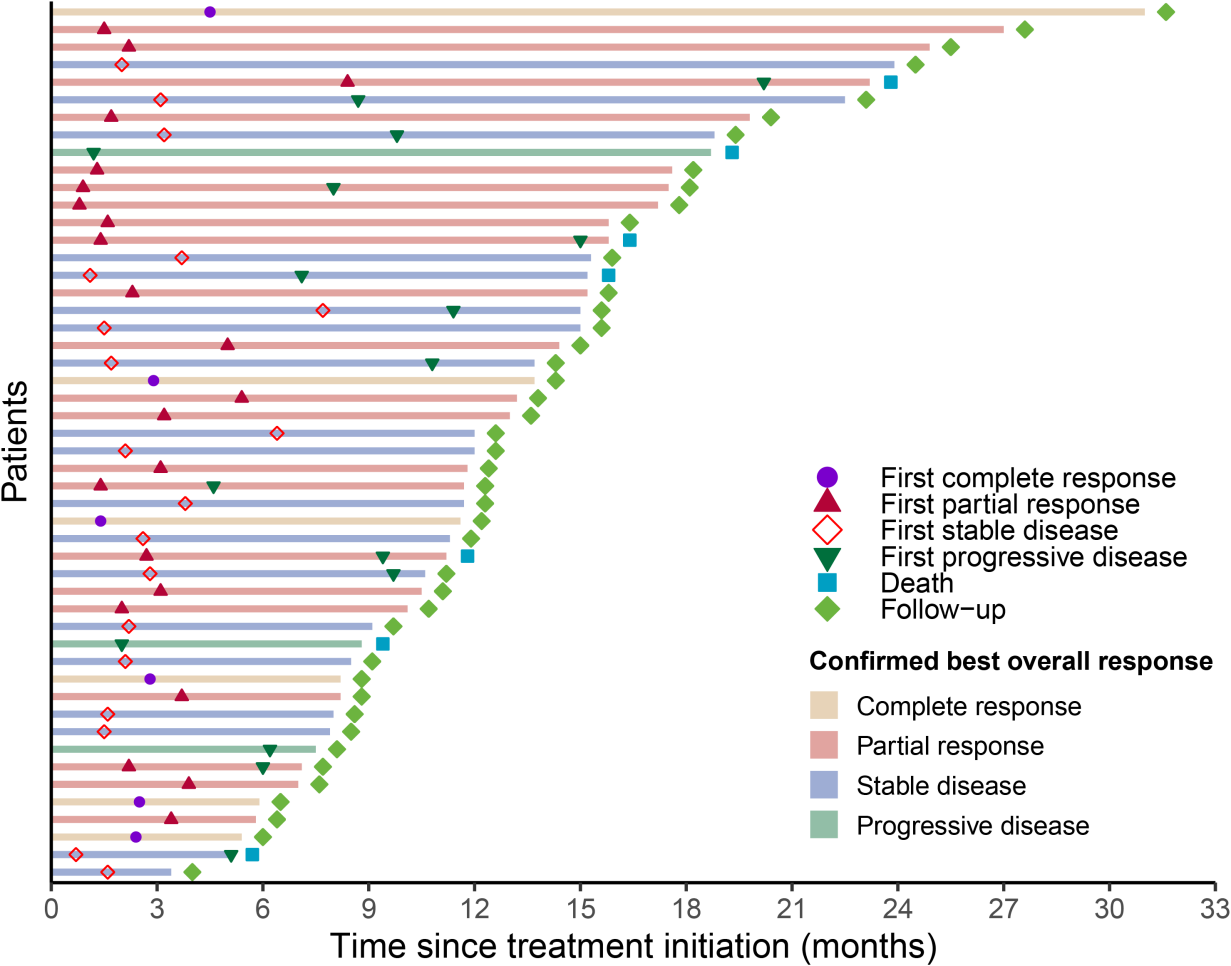
Progression events and duration of response according to RECIST 1.1.

The duration of treatment and timing of response for each evaluable patient in the cadonilimab plus TP group are illustrated in the swim-lane plot (**Figure 3**). Each horizontal bar represents an individual patient, with bar length corresponding to the time from treatment initiation to the last follow-up or death. The bar color indicates the confirmed BOR as assessed per RECIST v1.1. Markers overlaid on each bar depict the timing of the first objective responses (CR or PR), the first SD, the first PD, and death. The first objective responses occurred rapidly: the median time to initial CR or PR was 2.45 months [interquartile range (IQR): 1.58–3.25]. At the data cutoff, 22 of 28 responders (78.6%) had not experienced disease progression and remained under follow-up.

**Figure 4:**
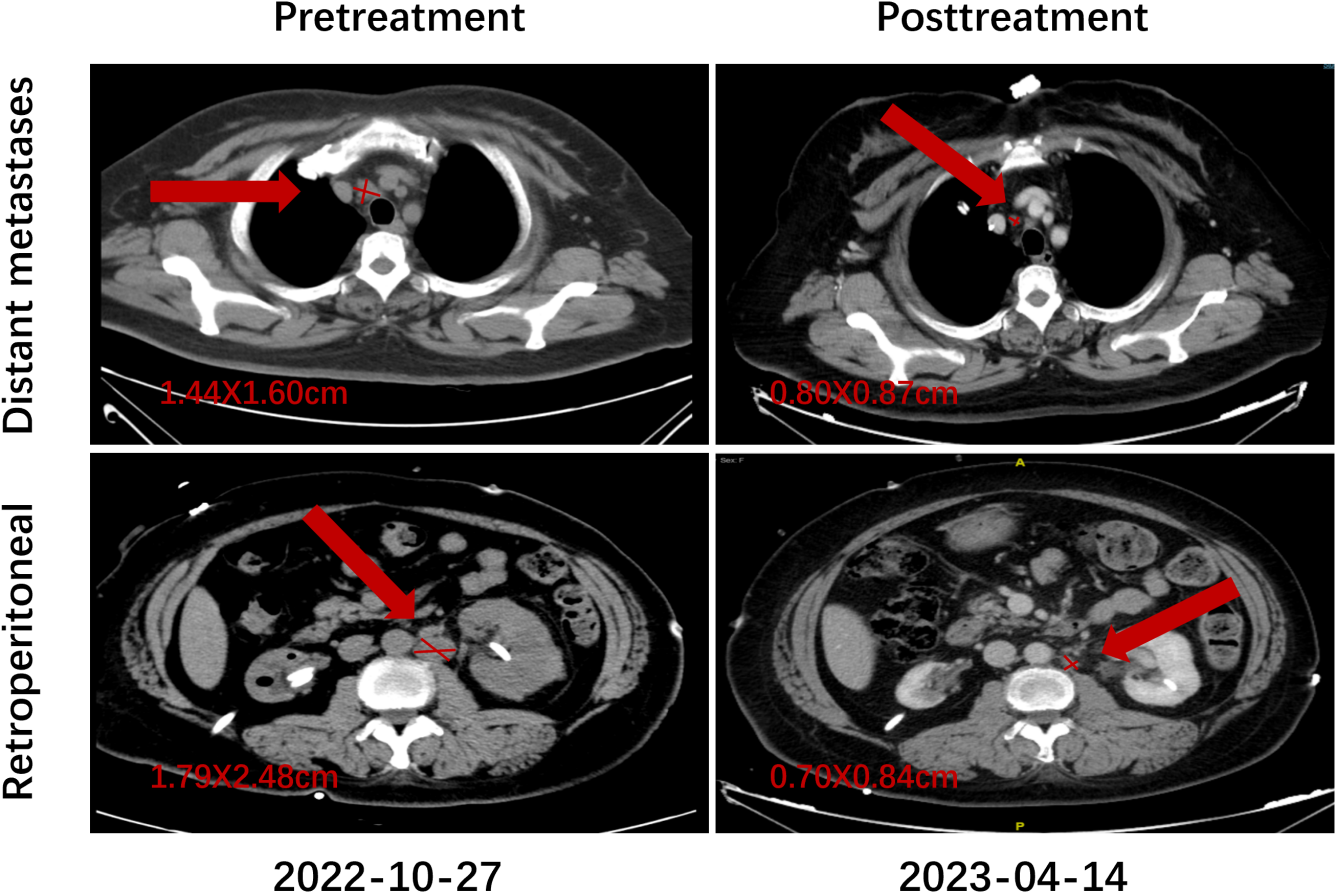
Representative imaging of treatment response in a patient who achieved CR.

Representative imaging findings are presented to visually illustrate treatment response. **Figure 4** shows the pretreatment and posttreatment radiologic images of paratracheal and para-aortic lymph node metastases in a patient who achieved a CR.

### 3.3 Efficacy

At the June 2025 data cutoff, follow-up duration estimated by the reverse Kaplan–Meier approach was 33.2 months overall (IQR: 12.2–35.2). By treatment group, median follow-up was 40.7 months (IQR: 17.6–40.4) in the TP group and 13.0 months (IQR: 8.6–15.8) in the cadonilimab plus TP group.

After two treatment cycles, a short-term efficacy assessment was conducted, and a numerically higher proportion of patients receiving cadonilimab plus TP achieved a CR (12.0% vs. 5.9%) and a PR (42.0% vs. 34.5%), whereas rates of SD (38.0% vs. 50.4%) and PD (8.0% vs. 9.2%) were correspondingly lower relative to TP alone (**Table 2**).

**Table 2:** Response evaluation after two cycles of treatment (*N* = 169).

Response	TP group (*n* = 119), *N* (%)	Cadonilimab plus TP group (*n* = 50), *N* (%)	*p*-value
CR	7 (5.9)	6(12.0)	0.208
PR	41 (34.5)	21 (42.0)	0.353
SD	60 (50.4)	19 (38.0)	0.140
PD	11 (9.2)	4(8.0)	0.795
ORR	48 (40.3)	27(54.0)	0.103
DCR	108 (90.8)	46(92.0)	0.795

**Figure 5:**
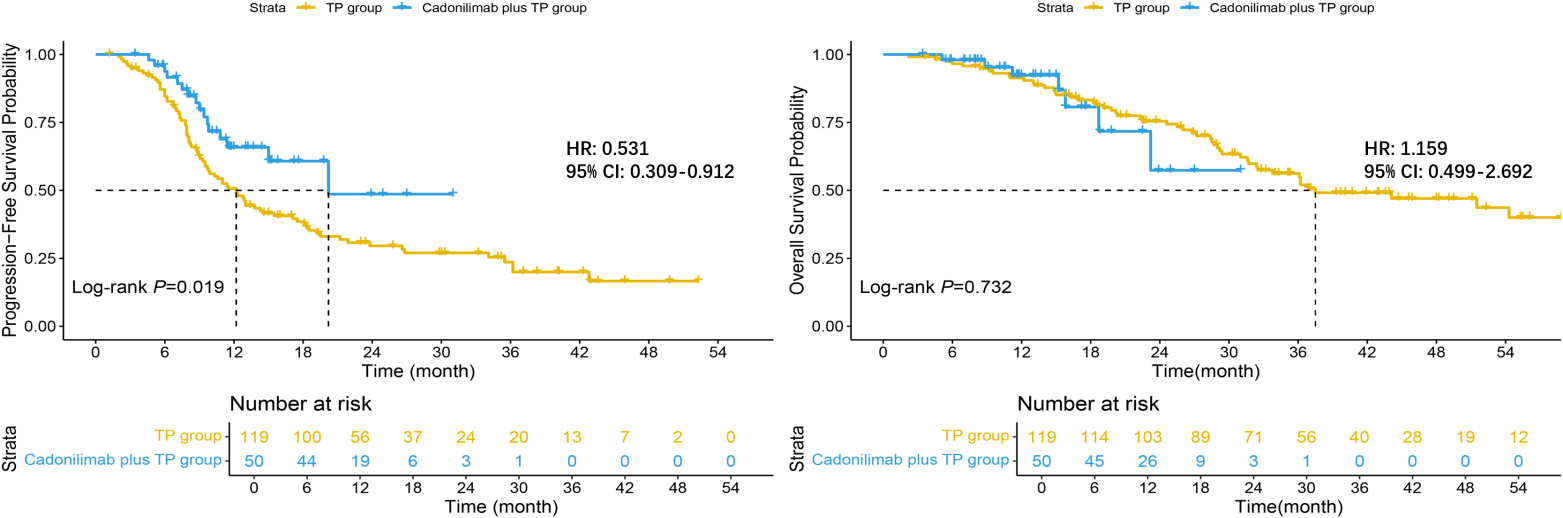
Kaplan–Meier curves for survival outcomes.

Among the 169 evaluable patients, the addition of cadonilimab produced a pronounced and statistically significant delay in tumor progression: mPFS was 20.2 months (95% CI: 15.0–NR) versus 12.2 months (95% CI: 9.7–17.1) with chemotherapy alone (log-rank *p* = 0.019). By contrast, a clear OS advantage had not yet emerged at the data cutoff; mOS was not reached in the cadonilimab plus TP group (95% CI: 23.2–NR) and was 37.5 months (95% CI: 32.4–NR) in the TP group, with overlapping Kaplan–Meier curves and a non-significant log-rank comparison (*p* = 0.732). These findings indicate that cadonilimab confers an early and durable PFS benefit, whereas a definitive OS benefit awaits longer follow-up (**Figure 5**).

At the landmark analysis, the cadonilimab plus TP yielded higher PFS rates than TP alone: 65.8% (95% CI: 52.6–82.5) versus 50.7% (95% CI: 42.3–60.8) at 12 months and 48.6% (95% CI: 29.0–81.5) versus 29.6% (95% CI: 21.9–39.9) at 24 months. OS rates were similarly high at 12 months—92.3% (95% CI: 84.3–100.0) with cadonilimab plus TP and 91.4% (95% CI: 86.4–96.6) with TP—but diverged at 24 months, with the investigational arm at 57.4% (95% CI: 33.5–98.2) versus 75.5% (95% CI: 67.8–84.0) for TP alone; the wide confidence interval in the cadonilimab cohort indicates limited events and underscores the need for longer follow-up to confirm any definitive OS benefit.

### 3.4 Treatment-related adverse events

TRAEs were common yet largely low−grade, indicating a manageable toxicity profile for both regimens (**Table 3**). The cadonilimab plus TP group showed higher incidences of hypoalbuminemia (46.0% vs. 27.7%), hypothyroidism (28.0% vs. 7.6%), pyrexia (32.0% vs. 11.8%), rash/itching (38.0% vs. 8.4%), constipation (58.0% vs. 31.9%), diarrhea (50.0% vs. 27.7%), and fatigue (74.0% vs. 45.4%), each reaching statistical significance (*p* < 0.05). Conversely, no significant between−group differences were observed for hematologic abnormalities such as anemia, leukopenia, neutropenia, or thrombocytopenia, or for elevations in alanine or aspartate aminotransferase (*p* > 0.05). Grade 3–5 TRAEs were infrequent across both cohorts; only severe leukopenia occurred less often with cadonilimab plus TP (8.0% vs. 21.0%, *p* = 0.041).

**Table 3:** Treatment-related adverse events (*N* = 169).

Adverse events	TP group (*n* = 119), *N* (%)		Cadonilimab plus TP group (*n* = 50), *N* (%)		*p*-value	
	All grades	Grades 3–5	All Grades	Grades 3–5	All grades	Grades 3–5
Anemia	88 (73.9)	7 (5.9)	41 (82.0)	7 (14.0)	0.261	0.122
White blood cell count decreased	73 (61.3)	25 (21.0)	29 (58.0)	4 (8.0)	0.685	0.041
Neutrophil count decreased	55 (46.2)	22 (18.5)	21 (42.0)	4 (8.0)	0.615	0.085
Platelet count decreased	64 (53.8)	1 (0.8)	23 (46.0)	0	0.356	1.000
Hypoalbuminemia	33 (27.7)	0	23 (46.0)	1 (2.0)	0.021	0.296
Alanine aminotransferase increased	29 (24.4)	1 (0.8)	15 (30.0)	0	0.446	1.000
Aspartate aminotransferase increased	18 (15.1)	1 (0.8)	14 (28.0)	0	0.051	1.000
Hypokalemia	27 (22.7)	3 (2.5)	13 (26.0)	2 (4.0)	0.644	0.633
Hypothyroidism	9 (7.6)	0	14 (28.0)	1 (2.0)	<0.001	0.296
Pyrexia	14 (11.8)	0	16 (32.0)	1 (2.0)	0.002	0.296
Immune-related myocarditis	0	0	1 (2.0)	1 (2.0)	0.296	0.296
Nausea/Vomiting	76 (63.9)	1 (0.8)	39 (78.0)	2 (4.0)	0.072	0.209
Decreased appetite	72 (60.5)	0	36 (72.0)	0	0.156	——
Rash/Itching	10 (8.4)	1 (0.8)	19 (38.0)	1 (2.0)	<0.001	0.505
Constipation	38 (31.9)	0	29 (58.0)	0	0.002	——
Diarrhea	33 (27.7)	0	25 (50.0)	0	0.005	——
Fatigue	54 (45.4)	1 (0.8)	37 (74.0)	1 (2.0)	<0.001	0.505

Among the 50 patients who received cadonilimab plus TP, two permanently discontinued cadonilimab due to grade ≥3 immune-related adverse events (irAEs). One patient developed grade 3 hypothyroidism, presenting with severe fatigue and bradycardia. Levothyroxine replacement therapy was initiated, resulting in gradual symptom improvement. Another patient experienced immune-related myocarditis, manifesting as anterior chest discomfort and significant fatigue. Laboratory evaluation revealed elevated creatine kinase (352 U/L), myoglobin (93.7 ng/mL), N-terminal pro-B-type natriuretic peptide (NT-proBNP: 318.8 pg/mL), and cardiac troponin T (77.95 pg/mL). Immunotherapy was discontinued, and the patient was referred to the cardiology department of a general hospital for further evaluation and management. Cardiac biomarkers were closely monitored during follow-up. Additionally, one patient temporarily interrupted cadonilimab in combination with chemotherapy due to the development of a rectovaginal fistula, which was surgically treated, allowing for subsequent resumption of treatment.

Overall, the addition of cadonilimab to paclitaxel-platinum chemotherapy increased several low−grade immune−related and gastrointestinal toxicities without exacerbating the burden of serious adverse events, supporting the regimen’s acceptable safety and tolerability.

### 3.5 Subgroup analysis

**Figure 6:**
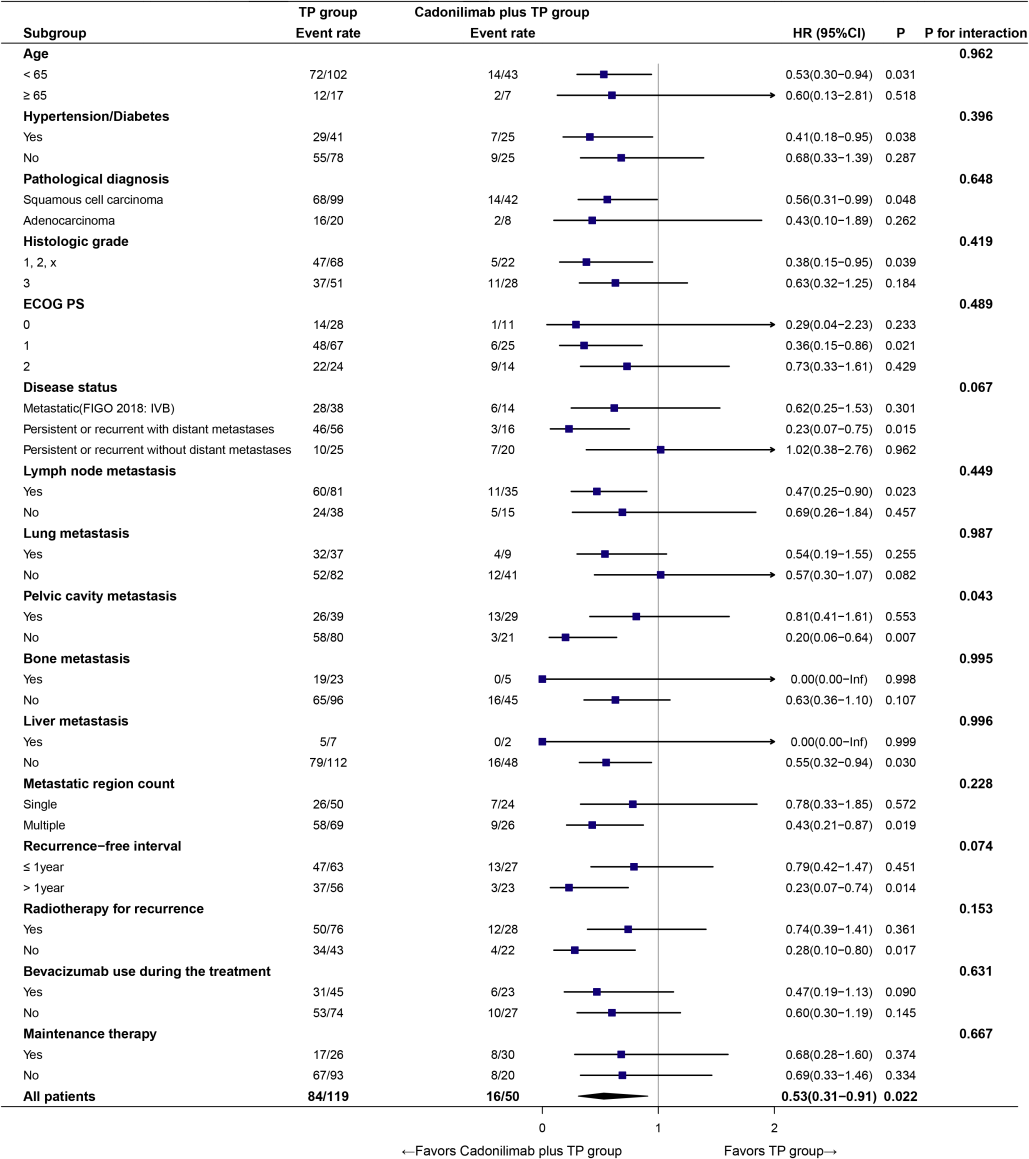
Subgroup analysis of PFS.

In prespecified subgroup analyses (**Figure 6**), cadonilimab plus TP prolonged PFS versus TP alone (HR = 0.53, 95% CI: 0.31–0.91; *p* = 0.022). The treatment effect consistently favored cadonilimab plus TP across most strata.

No significant treatment–subgroup interactions were detected for age, hypertension/diabetes, pathological diagnosis, histologic grade, ECOG PS, disease status, lymph node or lung/bone/liver metastasis, number of metastatic regions, recurrence−free interval, radiotherapy for recurrence, bevacizumab use during the treatment, or maintenance therapy (all *p* for interaction >0.05), except for pelvic cavity metastasis (*p* for interaction = 0.043). In that subgroup, patients without pelvic involvement derived a marked benefit (HR = 0.20, 95% CI: 0.06–0.64).

These findings suggest a broadly consistent benefit across diverse clinical subpopulations, with a potentially greater effect in the absence of pelvic cavity metastasis.

### 3.6 Prognostic factor analysis

In the univariate analysis (**Table 4**), an ECOG PS of 2 was significantly associated with inferior PFS (HR = 2.367, 95% CI: 1.535–3.651; *p* < 0.001). Higher tumor burden, including multiple metastatic regions (HR = 1.894, 95% CI: 1.244–2.884; *p* = 0.003) and lung metastasis (HR = 1.688, 95% CI: 1.120–2.544; *p* = 0.012), was also associated with poorer outcomes. Maintenance therapy was significantly associated with prolonged PFS (HR = 0.512, 95% CI: 0.325–0.806; *p* = 0.004), particularly among patients who received maintenance therapy for at least 1 year (HR = 0.217, 95% CI: 0.068–0.688; *p* = 0.009). A recurrence-free interval longer than 1 year was also associated with better PFS (HR = 0.659, 95% CI: 0.441–0.986; *p* = 0.043). Response evaluation after two cycles of treatment was a prognostic factor: patients achieving CR or PR after two cycles showed significantly improved PFS compared to those with SD (HR = 1.953, 95% CI: 1.270–3.004; *p* = 0.002). Additionally, first-line cadonilimab plus paclitaxel-platinum (± bevacizumab) reduced the hazard of progression by approximately 47% versus chemotherapy alone (HR = 0.531, 95% CI: 0.309–0.912; *p* = 0.022).

**Table 4:** Univariate and multivariate Cox regression analysis of PFS and OS in all patients (*N* = 169).

Variable	PFS				OS			
	Univariate analysis		Multivariate analysis		Univariate analysis		Multivariate analysis	
	HR (95%CI)	*p-*value	HR (95% CI)	*p-*value	HR (95% CI)	*p-*value	HR (95% CI)	*p-*value
**Age** (≥65 vs. <65)	1.045 (0.592–1.843)	0.879			0.993 (0.471–2.097)	0.986		
**Diabetes** (Yes vs. No)	1.062 (0.650–1.736)	0.810			0.831 (0.421–1.639)	0.593		
**Hypertension** (Yes vs. No)	0.944 (0.613–1.454)	0.794			0.846 (0.477–1.499)	0.566		
**ECOG PS** (2 vs. ≤1)	2.367 (1.535–3.651)	<0.001	2.284 (1.423–3.667)	<0.001	3.021 (1.783–5.120)	<0.001	3.592 (2.005–6.436)	<0.001
**Disease status**								
Persistent or recurrent with distant metastases vs. Metastatic (FIGO 2018: IVB)	0.985 (0.635–1.528)	0.947			0.827 (0.466–1.467)	0.515		
Persistent or recurrent without distant metastases vs. Metastatic (FIGO 2018: IVB)	0.635 (0.354–1.137)	0.126			0.526 (0.238–1.166)	0.114		
**Pathological diagnosis** (Adenocarcinoma vs. Squamous cell carcinoma)	1.090 (0.654–1.818)	0.741			1.139 (0.592–2.195)	0.696		
**Histologic grade** (3 vs. 1,2,x)	1.183 (0.798–1.755)	0.403			1.198 (0.722–1.990)	0.484		
**Metastatic region count** (Multiple vs. Single)	1.894 (1.244–2.884)	0.003	1.562 (0.941–2.592)	0.085	2.101 (1.195–3.695)	0.010	2.020 (1.035–3.942)	0.039
**Lymph node metastasis** (Yes vs. No)	1.315 (0.853–2.028)	0.215			1.486 (0.844–2.618)	0.170		
**Lung metastasis** (Yes vs. No)	1.688 (1.120–2.544)	0.012	1.784 (1.071–2.973)	0.026	1.551 (0.925–2.603)	0.096		
**Pelvic cavity metastasis** (Yes vs. No)	1.193 (0.796–1.789)	0.393			1.039 (0.609–1.773)	0.889		
**Bone metastasis** (Yes vs. No)	1.104 (0.669–1.821)	0.699			2.106 (1.148–3.863)	0.016	1.426 (0.715–2.843)	0.314
**Liver metastasis** (Yes vs. No)	0.894 (0.363–2.204)	0.808			2.159 (0.858–5.431)	0.102		
**Radiotherapy for recurrence** (Yes vs. No)	0.924 (0.616–1.385)	0.702			1.061 (0.629–1.789)	0.825		
**Bevacizumab use during the treatment** (Yes vs. No)	0.867 (0.577–1.302)	0.492			1.049 (0.611–1.802)	0.862		
**Maintenance therapy** (Yes vs. No)	0.512 (0.325–0.806)	0.004	0.746 (0.443–1.254)	0.269	0.737 (0.397–1.367)	0.332		
**1 year of maintenance therapy** (Yes vs. No)	0.217 (0.068–0.688)	0.009	0.330 (0.096–1.132)	0.078	0.042 (0.001–1.753)	0.096		
**Recurrence-free interval** (>1 year vs. ≤1 year)	0.659 (0.441–0.986)	0.043	0.662 (0.400–1.094)	0.107	0.556 (0.328–0.944)	0.030	0.736 (0.398–1.363)	0.330
**Response evaluation after 2 cycles of treatment** (SD vs. CR+PR)	1.953 (1.270–3.004)	0.002	1.691 (1.089–2.628)	0.019	2.066 (1.137–3.756)	0.017	1.622 (0.875–3.004)	0.124
**Number of treatment cycles** (≥6 cycles vs. <6 cycles)	0.759 (0.506–1.138)	0.182			1.023 (0.612–1.711)	0.931		
**Therapy** (Cadonilimab plus TP vs. TP)	0.531 (0.309–0.912)	0.022	0.599 (0.328–1.094)	0.095	1.159 (0.499–2.692)	0.732		

Bold values are shown solely for visual clarity and do not carry any statistical implication

In multivariate analysis, ECOG PS = 2 (HR = 2.28, 95% CI: 1.42–3.67; *p* < 0.001), lung metastasis (HR = 1.78, 95% CI: 1.07–2.97; *p* = 0.026), and lack of early objective response (SD vs. CR/PR; HR = 1.69, 95% CI: 1.09–2.63; *p* = 0.019) were identified as independent adverse prognostic factors.

OS analyses yielded a pattern similar to that observed for PFS. On univariate assessment, ECOG PS = 2 remained the strongest adverse factor (HR = 3.021, 95% CI: 1.783–5.120; *p* < 0.001), while multiple metastatic sites (HR = 2.101, 95% CI: 1.195–3.695; *p* = 0.010), bone metastasis (HR = 2.106, 95% CI: 1.148–3.863; *p* = 0.016), recurrence−free interval ≤ 1 year (HR = 0.556, 95% CI: 0.328–0.944; *p* = 0.030), and early SD versus objective response after two cycles (HR = 2.066, 95% CI: 1.137–3.756; *p* = 0.017) were also significant. Multivariate modeling confirmed ECOG PS = 2 (HR = 3.592, 95% CI: 2.005–6.436; *p* < 0.001) and multiple metastatic regions (HR = 2.020, 95% CI: 1.035–3.942; *p* = 0.039) as independent predictors of poorer OS.

## 4 Discussion

Clinical deployment of bispecific antibodies in solid malignancies has accelerated. Moreover, an ongoing phase III study (NCT05446883) is evaluating QL1706, a PD-1/CTLA-4 bispecific with functional similarity to cadonilimab, as first-line treatment with paclitaxel-platinum ± bevacizumab for patients with r/m CC.

In our real-world study, cadonilimab combined with standard chemotherapy conferred survival benefits with an acceptable safety profile in patients with p/r/m CC. The key efficacy endpoints: PFS and the observed pattern of adverse events were largely consistent with findings from the AK104–303 trial.

Cadonilimab plus TP produced a clear PFS advantage over TP alone (mPFS: 20.2 vs. 12.2 months; log-rank *p* = 0.019), with higher 12- and 24-month PFS rates (65.83% and 48.62% vs. 50.71% and 29.57%, respectively). The direction of benefit is consistent with the AK104–303 trial, in which cadonilimab combined with chemotherapy also outperformed chemotherapy alone (mPFS: 12.7 vs. 8.1 months). However, the OS data remain immature at the current follow−up, and no statistically significant difference has been observed between groups to date. Notably, the mPFS in both groups of our real−world cohort exceeded that reported in AK104−303. This difference may reflect the frequent use of individualized local radiotherapy for metastatic lesions (63.9% in the TP group and 56.0% in the cadonilimab plus TP group) and the adoption of more diverse maintenance strategies in routine practice. By contrast, maintenance in AK104−303 was limited to cadonilimab with or without bevacizumab in the investigational arm, and the trial report did not specify whether local radiotherapy to metastatic sites was permitted or utilized (11). Extensive data (20–24) support integrating local radiotherapy (including metastasis−directed SBRT) with systemic therapy as a reasonable strategy when overall tumor burden has been cytoreduced or is confined to a limited number of lesions.

In terms of safety, the most common adverse event was anemia (82.0%), followed by other known chemotherapy-related toxicities, such as nausea/vomiting, fatigue, decreased appetite, white blood cell count decreased, and constipation, all of which were manageable. Notably, irAEs such as rash/itching (38.0%), pyrexia (32.0%), and hypothyroidism (28.0%) occurred at relatively high frequencies, highlighting the importance of early recognition and timely management of irAEs to ensure treatment continuity.

We further identified that an ECOG PS of 2 consistently emerged as the strongest adverse prognostic factor for both PFS and OS in univariate and multivariate analyses. A high tumor burden, particularly the presence of lung metastasis and involvement of multiple metastatic sites, was also independently associated with poorer outcomes. Consistent with prior evidence, a greater baseline tumor burden portends inferior outcomes in advanced CC (25). Across multicenter cohorts (26), multi−organ or multisite metastases confer significantly shorter survival than single−site disease, whereas “low−burden” presentations, such as ≤5 lesions or nodal−only involvement, are independently associated with more favorable outcomes. In contrast, achieving an early objective response (CR/PR vs. SD) after two cycles of treatment was significantly associated with improved survival, emphasizing the prognostic relevance of early treatment response.

Moreover, maintenance therapy, particularly when administered for ≥1 year, was significantly linked to prolonged PFS, suggesting its potential role in sustaining clinical benefit. A multicenter real−world study (27) of first−line maintenance therapy, including PD−1 inhibitors, targeted agents, or both, reported significant gains in both PFS and OS; notably, >8 cycles correlated with additional PFS/OS extension, supporting a duration–response relationship that aligns with a ≥1-year strategy. A recurrence-free interval longer than 1 year prior to treatment initiation was also correlated with more favorable PFS.

Mechanistically, cadonilimab leverages concurrent PD−1 and CTLA−4 blockade to reinvigorate effector T−cell activity and augment T−cell priming while mitigating Treg−mediated suppression (28, 29); its tetravalent, Fc−silent architecture confers preferential avidity in dual−high PD−1/CTLA−4 tumor milieus and minimizes Fc−mediated toxicities (30), thereby supporting rational combinations with chemotherapy, anti−angiogenic agents, and local modalities (31–33).

Building on dual-pathway immune activation and tumor microenvironment remodeling, combination strategies with chemotherapy, anti-angiogenic therapy, and local modalities (e.g., radiotherapy or interventional procedures) show promise for further clinical development. Mechanistic and translational studies are needed to clarify how these regimens enhance efficacy and overcome resistance through pathway blockade, vascular normalization, and immune-contexture reshaping, thereby informing treatment sequencing and biomarker-guided patient selection.

Nonetheless, this research has a few limitations, such as a limited sample size, a relatively brief follow-up period, and being conducted at a single center. Moreover, PD−L1 tumor expression status was missing in a subset of patients and therefore was not incorporated into subgroup analyses or related exploratory evaluations. Future investigations with larger, multicenter real−world cohorts and complete biomarker profiling are warranted to corroborate these findings.

## 5 Conclusions

In real-world single-center practice, first-line cadonilimab plus paclitaxel-platinum ± bevacizumab improved PFS and increased ORR versus chemotherapy alone, with manageable toxicity and consistent subgroup effects, supporting frontline use and prompting further study of maintenance therapy.

## Data Availability

The raw data supporting the conclusions of this article will be made available by the authors, without undue reservation.
